# Durable Hematologic Response in Therapy-Related Myelodysplastic Syndrome During Nivolumab Treatment for Metastatic Melanoma: A Case Report

**DOI:** 10.3390/curroncol33080476

**Published:** 2026-08-13

**Authors:** Fathima Nashfa M. Hamza, Fatima Eltayeb, Mohammed Al Katari, Mohammed F. K. Ibrahim

**Affiliations:** 1 Regional Cancer Care Northwest, Thunder Bay Regional Health Sciences Centre, Thunder Bay, ON P7B 6V4, Canada; fathima.mhamza@gmail.com (F.N.M.H.); mohammed.al-katari@tbh.net (M.A.K.); 2 Wythenshawe Hospital, Manchester University NHS Foundation Trust, Southmoor Road, Wythenshawe, Manchester M23 9LT, UK; fatima.eltayeb@nhs.net; 3 Division of Clinical Sciences, Northern Ontario School of Medicine University, Thunder Bay, ON P7B 5E1, Canada

**Keywords:** immunotherapy, melanoma, Myelodysplastic syndrome, nivolumab, checkpoint inhibitor, case report

## Abstract

Immunotherapy is the standard first-line palliative treatment for metastatic melanoma; however, its role in myelodysplastic syndrome (MDS) remains uncertain. We report a case in which nivolumab, administered for metastatic melanoma, was associated with a marked improvement in concurrent treatment-related MDS. A 67-year-old man with a history of oligosecretory multiple myeloma developed transfusion-dependent MDS following autologous stem cell transplantation, requiring twice-weekly blood transfusions. He subsequently developed metastatic melanoma and was treated with single agent nivolumab to minimize treatment-related toxicity. Nivolumab effectively controlled the melanoma, and during treatment the patient experienced a durable hematologic response with sustained transfusion independence. At approximately two years of follow up, he remained transfusion-independent with stable peripheral blood counts, supporting the durability of the hematologic response. Although checkpoint inhibitors are not established therapies for therapy-related MDS and a causal relationship cannot be confirmed from a single case, the timing and sustained nature of the response are noteworthy. The patient experienced a significant improvement in quality of life with minimal adverse effects. This case adds to the limited literature on checkpoint inhibition in MDS and supports further investigation of its potential effects on hematologic recovery.

## Simple Summary

Immunotherapy has transformed the treatment of several cancers, including advanced melanoma, but its role in myelodysplastic syndrome (MDS) remains unclear. We report the case of a 67-year-old man who had previously been treated for multiple myeloma with chemotherapy, radiotherapy, and autologous stem cell transplantation. He later developed therapy-related MDS, resulting in severely low blood counts and the need for frequent blood transfusions. Several months later, he was diagnosed with metastatic melanoma and started on nivolumab—an immunotherapy drug—to treat the melanoma. After starting nivolumab, his blood count gradually improved and he no longer required blood transfusions. This improvement was sustained for 2 years during long-term follow-up. Although it is not possible to confirm that nivolumab was responsible for the hematologic recovery, the timing and prolonged duration of the response are noteworthy. Further studies are needed to understand whether similar responses may occur in other patients with therapy-related MDS.

## 1. Introduction

Therapy-related myelodysplastic syndrome (t-MDS) is a recognized late complication of cytotoxic therapy and is associated with limited treatment options and poor clinical outcomes [1,2]. Patients with multiple myeloma who undergo high-dose chemotherapy followed by autologous stem cell transplantation are at increased risk of developing t-MDS or therapy-related acute myeloid leukemia (AML), as recently reported by Yalniz et al. [3]. Despite increasing recognition of this complication, evidence-based recommendations for surveillance and management remain limited [2].

Nivolumab is a programmed cell death protein-1 (PD-1) immune checkpoint inhibitor widely used in the treatment of metastatic melanoma and other advanced malignancies [4]. However, its effects on therapy-related MDS remain poorly understood.

We describe a patient with concurrent metastatic melanoma and transfusion-dependent therapy-related MDS who developed a durable hematologic response with sustained transfusion independence while receiving nivolumab for metastatic melanoma.

## 2. Case Presentation

A 67-year-old Caucasian man was diagnosed with a T3 solitary plasmacytoma in January 2021 following CT-guided core biopsy. Bone marrow biopsy at the time showed no evidence of plasma cell dyscrasia, with no detectable monoclonal protein or other sites of plasmacytoma involvement. He received radiotherapy in March 2021 (20 fractions; total dose, 4000 cGy), which was complicated by radiation pneumonitis and a left lower extremity deep vein thrombosis. His other medical history included hypertension and diet-controlled type 2 diabetes mellitus.

In October 2021, surveillance PET-CT identified a new lesion, prompting further investigation. Repeat bone marrow aspiration and biopsy confirmed progression to non-secretory multiple myeloma, with <5% plasma cells and monoclonal B-cell lymphocyte proliferation. He was treated with four cycles of cyclophosphamide, bortezomib, and dexamethasone, together with monthly zoledronic acid. Autologous stem cell transplantation was performed in February 2022. Lenalidomide maintenance therapy was started in June 2022, with filgrastim support.

In December 2023, investigation of severe thrombocytopenia prompted bone marrow aspiration and biopsy. Histopathological examination demonstrated dysplastic trilineage hematopoiesis, rare ring sideroblasts, and approximately 7% myeloid blasts. Conventional cytogenetic analysis revealed a normal male karyotype, while next-generation sequencing identified an EZH2 mutation with a variant allele frequency (VAF) of 45%. Based on the morphologic, cytogenetic, and molecular findings, a diagnosis of therapy-related myelodysplastic syndrome was established. Lenalidomide and rivaroxaban were discontinued.

Despite discontinuation of lenalidomide, there was no sustained recovery in peripheral blood counts over the following months. He developed profound pancytopenia, requiring frequent platelet and packed red blood cell transfusions—initially twice weekly and subsequently weekly.

Repeat bone marrow examinations performed in January and February 2024 demonstrated resolution of the previously increased blast population but persistent marked marrow hypocellularity with residual erythroid dysplasia and rare ring sideroblasts. The differential diagnosis at that stage included hypocellular MDS, drug-induced marrow suppression related to prior lenalidomide exposure, and aplastic anemia; however, the patient remained transfusion dependent with ongoing severe cytopenias.

**Figure 1:**
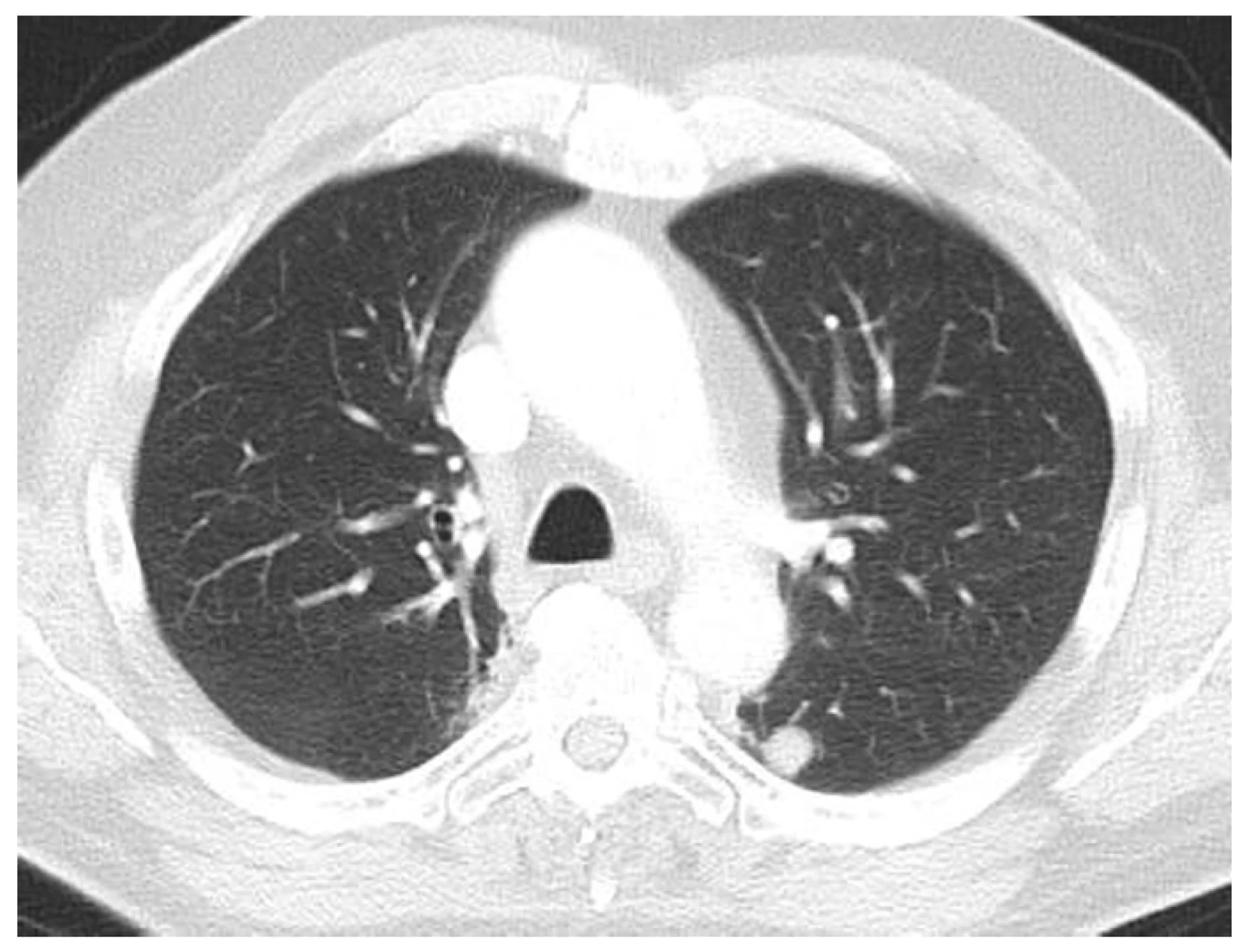
Axial chest CT showing a 1.2 cm nodule in the left lower lobe of the lung.

**Figure 2:**
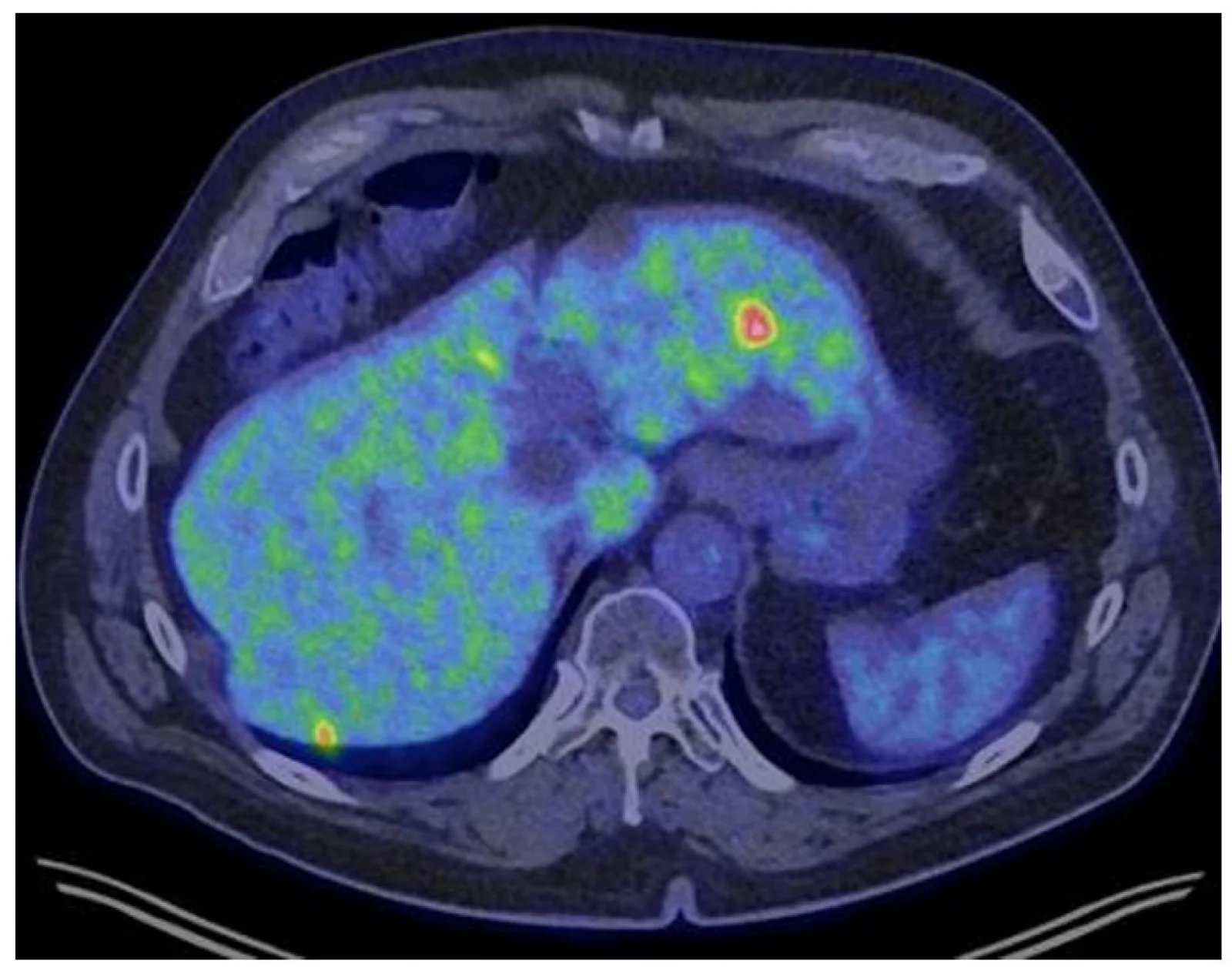
PET scan showing multiple hypermetabolic lesions in the liver consistent with metastatic disease.

Concurrent CT imaging in December 2023 revealed a 1.2 cm pulmonary nodule in the left lower lobe (Figure 1). Subsequent PET/CT in February 2024 demonstrated widespread metastatic disease involving the liver, lungs, and bone, as well as the mediastinal and hilar lymph nodes (Figure 2). Liver biopsy confirmed metastatic melanoma. Given his impaired bone marrow reserve, single-agent nivolumab was initiated instead of combination nivolumab–ipilimumab to minimize treatment-related toxicity. Molecular analysis identified a BRAF mutation, offering an alternative treatment option if immunotherapy failed.

**Figure 3:**
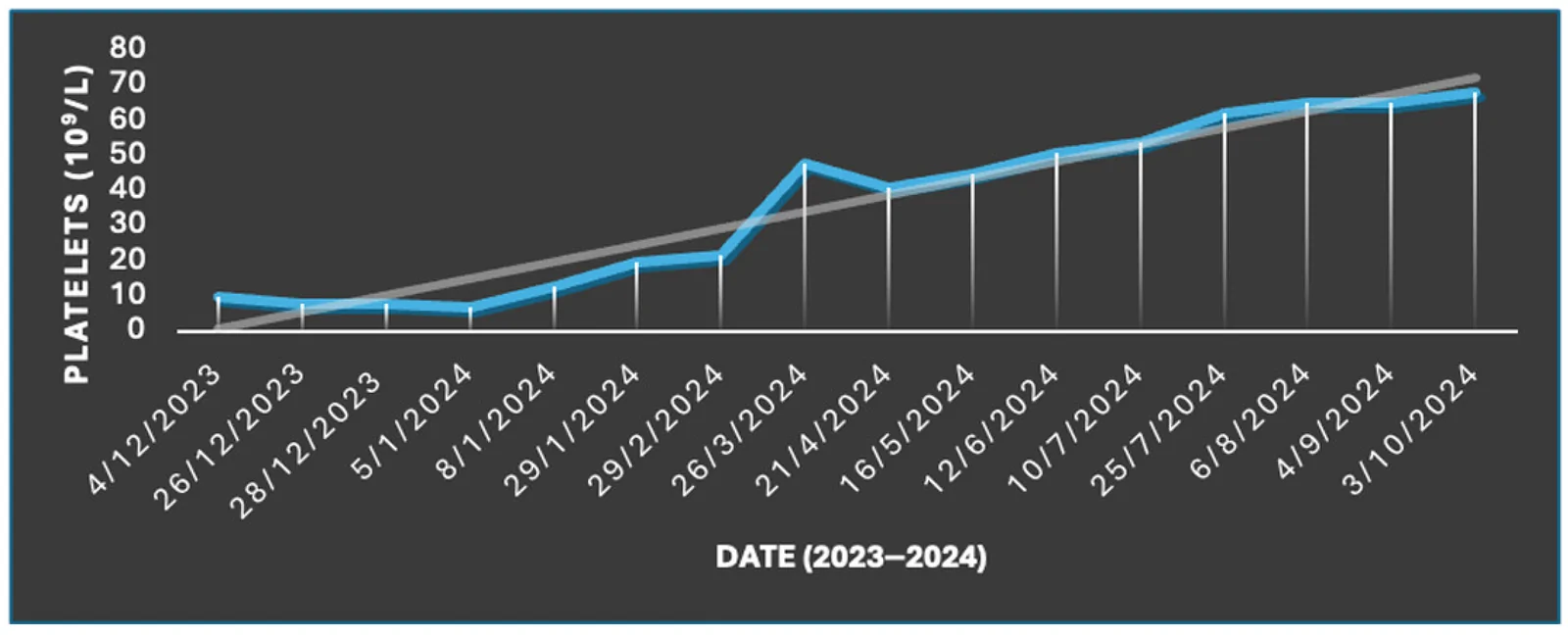
Trend in platelet counts before and after initiation of nivolumab therapy. Nivolumab was started in April 2024, after which platelet transfusions were discontinued. A steady upward trend is observed post-therapy.

**Figure 4:**
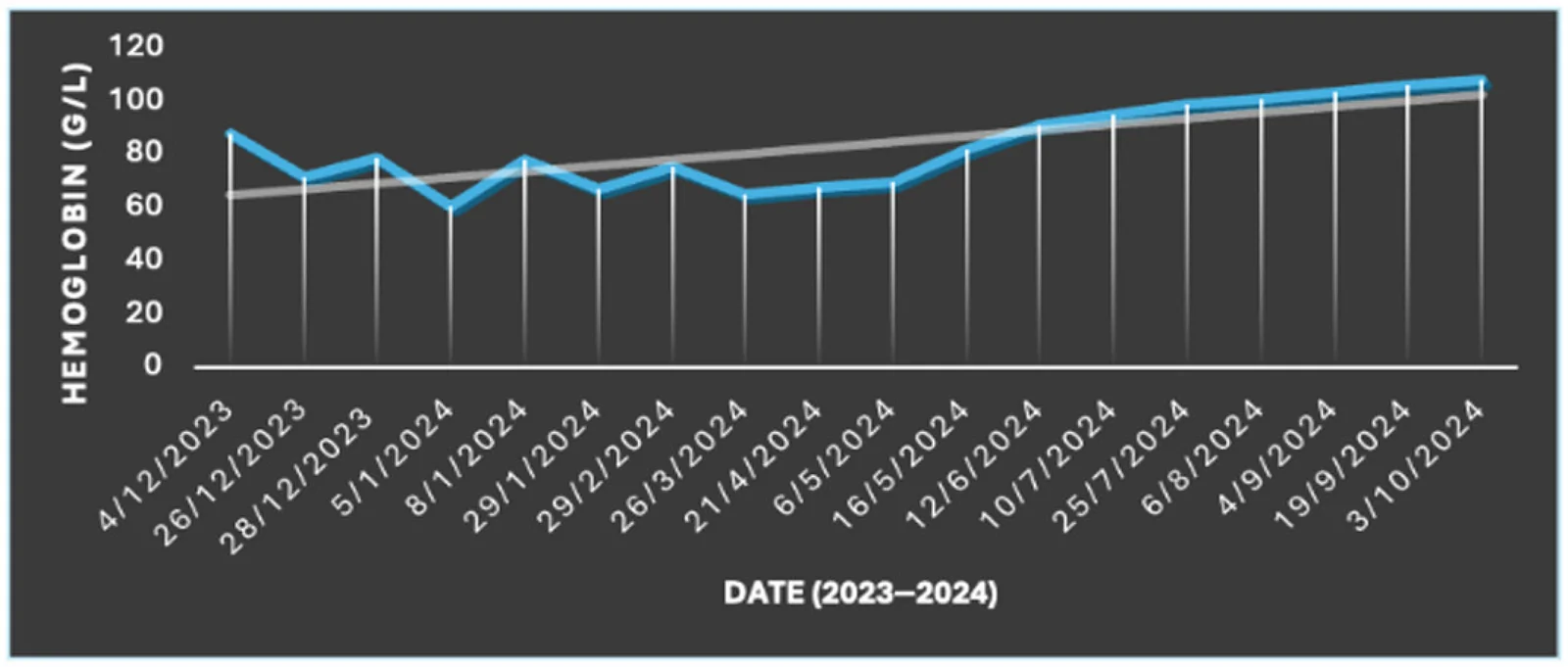
Trend in hemoglobin counts before and after initiation of nivolumab therapy. Nivolumab was started in April 2024, and packed red blood cell transfusions were discontinued in May 2024. Hemoglobin levels show a steady upward trend post-therapy.

**Figure 5:**
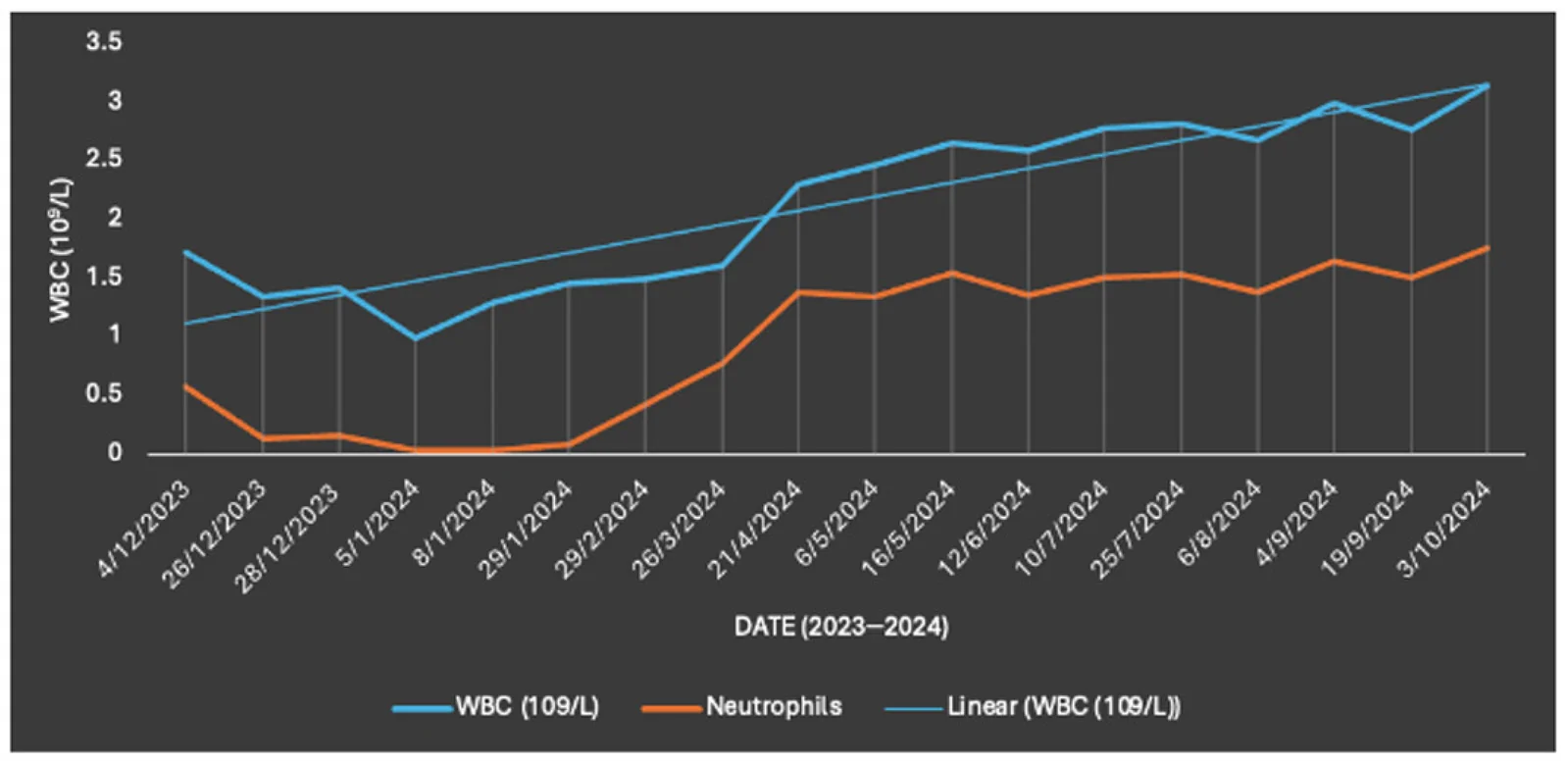
Trend in white blood cell (WBC) and neutrophil counts before and after initiation of nivolumab therapy. Nivolumab was started in April 2024. Both WBC and neutrophil counts show a steady upward trend post-therapy.

On 19 April 2024, the patient began nivolumab 3 mg/kg (240 mg) every two weeks. Following initiation of therapy, platelet, hemoglobin and leukocyte counts began to improve considerably. The patient received his final packed red blood cell transfusion on 6 May 2024. Thereafter, he remained transfusion-independent. Over the subsequent weeks, hematologic parameters continued to recover, with platelet counts increasing from 48 to 68 × 10^9^/L, hemoglobin from 65 to 108 g/L, and white blood cell counts from 1.61 to 3.15 × 10^9^/L. By June 2024, the patient’s Edmonton Symptom Assessment System (ESAS) scores were 0 across all domains, reflecting complete resolution of symptom burden (Figure 3, Figure 4 and Figure 5).

By October 2024, after seven cycles of nivolumab, the patient demonstrated significant clinical improvements. He remained transfusion-independent, with stable complete blood counts and normal thyroid, hepatic, and renal function. The patient also reported significant improvements in his quality of life; energy levels; appetite; and physical symptoms, such as rashes, nausea, and vomiting. Bisphosphonate therapy was initiated for management of osseous metastases.

At the most recent follow up in May 2026, the patient remained transfusion-independent with stable peripheral blood counts and no further transfusion requirements. Progress notes described sustained hematologic recovery while metastatic melanoma remained radiographically stable on nivolumab therapy. He continued first-line palliative nivolumab, with a planned treatment break of 4 to 6 weeks. Hematological parameters throughout the clinical course are summarized in Table 1.

## 3. Discussion

This case describes a patient with transfusion-dependent therapy-related myelodysplastic syndrome (t-MDS) and metastatic melanoma who experienced a durable hematologic response during treatment with nivolumab for melanoma. To our knowledge, similar cases have rarely been reported in the literature.

The patient’s therapy-related MDS most likely arose following prior exposure to cytotoxic therapy (MDS-pCT), a rare but aggressive secondary MDS subtype. Prognosis is poor, with median survival of 8–10 months and a 5-year survival of only 10%, compared to approximately 31% in primary MDS [2]. This patient’s prior cyclophosphamide, bortezomib, and dexamethasone therapy, moderate-dose radiotherapy, and autologous stem cell transplantation demonstrates recognized risk factors for MDS [1,2,3]. Reported incidence of MDS-pCT is approximately 3.4% in multiple myeloma, 10% in non-Hodgkin lymphoma, and 8.2% in chronic lymphocytic leukemia [2]. Unlike most therapy-related MDS cases, where >90% have cytogenetic abnormalities [5], his karyotype was normal.

Our patient’s subsequent bone marrow examinations demonstrated increasingly hypocellular marrow, broadening the differential diagnosis to include drug-induced marrow suppression and aplastic anemia. These later findings did not necessarily exclude MDS, particularly given the initial trilineage dysplasia, approximately 7% myeloid blasts, and EZH2 mutation. Rather, they reflect the complexity of marrow failure in a heavily treated patient. Importantly, despite these evolving marrow findings, the patient continued to have severe pancytopenia and remained transfusion-dependent until nivolumab was initiated.

Lenalidomide, which was used as post-transplant maintenance in this case, has been associated with higher rates of secondary myelodysplasia [6]. However, causality is difficult to prove, as these patients often also receive high-dose chemotherapy and transplantation, both of which are independent MDS risk factors [7]. Autologous transplantation itself is linked to late-onset MDS and acute myeloid leukemia, major causes of post-transplant morbidity and mortality [7].

An important consideration is whether the hematologic improvement was related to nivolumab or represented delayed recovery after discontinuation of lenalidomide. Lenalidomide can cause significant myelosuppression, particularly thrombocytopenia and neutropenia, and its discontinuation may be followed by recovery of peripheral blood counts. In this patient, however, lenalidomide had been stopped approximately four months before nivolumab was started. During this interval, he remained profoundly pancytopenic and continued to require regular platelet and packed red blood cell transfusions, without sustained hematologic recovery. Other possible contributors include previous filgrastim use, delayed recovery following autologous stem cell transplantation, and natural fluctuation in the course of therapy-related MDS. However, transplantation had occurred more than two years before nivolumab was initiated, and filgrastim had been used earlier in the clinical course. The subsequent improvement across all three hematopoietic cell lines and sustained transfusion independence for two years after nivolumab initiation represent a notable temporal association. Nevertheless, these factors cannot be fully separated in a single case, and a direct causal effect of nivolumab cannot be established.

An additional limitation is that an IPSS-R or IPSS-M score was not documented at the time of diagnosis of MDS. As complete data required for formal risk stratification were unavailable, these scores could not be assigned retrospectively.

There is currently no established standard of treatment for MDS-pCT. Although it is associated with prior cytotoxic therapy, its molecular features often overlap with those of de novo MDS [8], making treatment decisions challenging. Patients with MDS-pCT generally have poor outcomes and remain at increased risk of progression to AML.

Although both ipilimumab and nivolumab are immune checkpoint inhibitors, they target different stages of the immune response. Ipilimumab blocks CTLA-4, primarily enhancing T-cell activation during the early priming phase within lymphoid tissues. In contrast, nivolumab targets PD-1, restoring the activity of exhausted T cells within peripheral tissues and the tumor microenvironment [9]. These differences may contribute to variations in both antitumor activity and immune-mediated effects outside the tumor.

The different hematologic response observed in our patient may therefore reflect the distinct mechanisms of PD-1 and CTLA-4 blockade rather than a general class effect of immune checkpoint inhibitors. However, this interpretation should be made with caution. Our patient did not receive ipilimumab because of concerns regarding treatment-related toxicity in the setting of severe pancytopenia and limited bone marrow reserve. As a result, it is not possible to determine whether a similar hematologic response would have occurred with CTLA-4 inhibition. The observed association with nivolumab is therefore hypothesis-generating and requires further evaluation in larger clinical studies.

Clinical evidence supporting the use of immune checkpoint inhibitors in hematologic malignancies remains limited. In a phase I multicenter trial, nivolumab was studied in patients with AML/MDS following allogeneic hematopoietic stem cell transplantation. Although nivolumab showed evidence of graft versus tumor activity, it was also associated with a significant risk of graft versus host disease (GVHD), particularly when treatment was started soon after transplantation [10,11]. A separate phase I/Ib trial evaluated ipilimumab in patients with relapsed hematologic malignancies after allogeneic stem cell transplantation. Durable responses were observed in a subset of patients, with a median overall survival of 28.2 months among those who received ipilimumab at 10 mg/kg [12].

Although these studies suggest that checkpoint inhibition may have activity in hematologic malignancies, both were conducted in the setting of allogeneic transplantation. This differs from our patient, who underwent autologous stem cell transplantation, where the risk and underlying mechanisms of GVHD are substantially different. These findings therefore cannot be directly applied to our case. Further studies are needed to determine the role of PD-1 blockade in therapy-related MDS and whether the timing of treatment influences the hematologic response.

The confirmation of MDS remission generally requires repeat bone marrow examination. In this case, a repeat bone marrow biopsy was not performed after the patient’s blood counts improved. As he remained clinically well, transfusion-independent, and had sustained improvement in his peripheral blood counts, follow-up was based on serial clinical assessment and laboratory monitoring. Therefore, the response described in this report reflects durable hematologic recovery rather than morphologically confirmed remission.

To our knowledge, this is the first reported case of a durable hematologic response with sustained transfusion independence in therapy-related MDS during nivolumab treatment for metastatic melanoma. Although causality cannot be established from a single case, the temporal relationship between nivolumab initiation and subsequent hematologic recovery is noteworthy. Alternative explanations, including delayed recovery following discontinuation of lenalidomide, cannot be excluded. However, the patient remained profoundly pancytopenic and transfusion-dependent after lenalidomide was discontinued, with no sustained improvement in peripheral blood counts before nivolumab was initiated.

A particular strength of this case is the prolonged follow-up. Nearly two years after starting nivolumab, the patient remains transfusion-independent with stable peripheral blood counts while continuing nivolumab treatment for metastatic melanoma. Although repeat bone marrow evaluation was not performed to confirm morphologic remission, the sustained hematologic response makes a transient recovery less likely and supports further investigation into the potential role of PD-1 blockade in selected patients with therapy-related MDS.

## 4. Conclusions

Transfusion-dependent therapy-related myelodysplastic syndrome is associated with limited treatment options and poor clinical outcomes. We describe a patient with therapy-related MDS and metastatic melanoma. He experienced a durable hematologic response with sustained transfusion independence while receiving single-agent nivolumab for metastatic melanoma. Although a causal relationship cannot be established from a single case and alternative explanations cannot be completely excluded, the prolonged hematologic recovery observed in this patient is noteworthy. As no follow-up bone marrow examination was performed, this case represents sustained peripheral hematologic recovery rather than morphologically confirmed MDS remission. This case adds to the limited literature on the potential effects of PD-1 blockade in therapy-related MDS and supports further investigation in selected patients.

## Figures and Tables

**Table 1:** Hematologic parameters during the clinical course.

Date	Clinical Stage	WBC (×10^9^/L)	Hemoglobin (g/L)	Platelets (×10^9^/L)	ANC (×10^9^/L)	Transfusion Status
10 October 2023	Before MDS diagnosis	5.43	115	79	3.85	No
4 December 2023 *	MDS diagnosis	1.72	88	10	0.57	Yes
5 February 2024	Before nivolumab (near nadir)	1	72	5	0.07	Yes
19 April 2024 ^†^	Nivolumab initiated, ongoing transfusion dependence (closest available CBC)	2.78	74	43	1.71	Yes
6 May 2024	Last PRBC transfusion	2.47	70	32	1.35	Final transfusion
23 May 2024	Early hematologic response	2.58	77	48	1.64	No
25 July 2024	Continued response	2.82	99	62		No
30 October 2024	After 7 cycles of nivolumab	3.42	110	71	1.98	No
May 2026	Latest follow up	Stable	Stable	Stable	Stable	No

* Bone marrow biopsy showed dysplastic trilineage hematopoiesis, approximately 7% myeloid blasts, rare ring sideroblasts, normal karyotype, and an EZH2 mutation (VAF 45%). ^†^ Closest available CBC to nivolumab initiation.

## Data Availability

The data that support the findings of this study are available from the corresponding author upon reasonable request.
